# Be Good to Yourself

**Published:** 1898-02

**Authors:** 


### Be Good to Yourself.

Think deliberately of the house you live in—your body. Make up your mind firmly not to abuse it. Eat nothing that will hurt it. Wear nothing that distorts or pains it. Do not overload it with victuals or drink or work. Give yourself regular and abundant sleep. Keep your body warmly clad. Do not take cold; guard yourself against it. If you feel the first symptoms, give yourself heroic treatment. Get into a fine glow of heat by exercise. This is the only body you will have in this world. Study deeply and diligently the structure of it, the laws that govern it, the pains and penalty that will surely follow a violation of every law of life and health.—*Medical and Surgical Reporter*.

### Conversational Pitfalls.

MISS MEADOWSWEET—Excuse my ignorance, but ought I to call you Mr. Squills or Dr. Squills?

THE DOCTOR—Oh, call me anything you like. Some of my friends call me an old fool!

MISS MEADOWSWEET—Ah, but that's people who know you intimately!—*London Punch*.

